# Robot-assisted radical prostatectomy significantly reduced biochemical recurrence compared to retro pubic radical prostatectomy

**DOI:** 10.1186/s12885-017-3439-6

**Published:** 2017-06-29

**Authors:** Tetsuya Fujimura, Hiroshi Fukuhara, Satoru Taguchi, Yuta Yamada, Toru Sugihara, Tohru Nakagawa, Aya Niimi, Haruki Kume, Yasuhiko Igawa, Yukio Homma

**Affiliations:** 1 Department of Urology, Graduate School of Medicine, The University of Tokyo, 7-3-1 Hongo, Bunkyo-ku, Tokyo, 113-8655 Japan; 2 Department of Urology, Japan Red Cross Hospital, 4-1-22 Hiroo, Shibuya-ku, Tokyo, Japan; 3 Department of Urology, Teikyo University, 2-11-1 Kaga, Itabashi-ku, Tokyo, Japan; 4 Department of Continence Medicine, Graduate School of Medicine, The University of Tokyo, 7-3-1 Hongo, Bunkyo-ku, Tokyo, 113-8655 Japan

**Keywords:** Oncological outcome, Retro pubic radical prostatectomy (RRP), Prostate cancer, And robot-assisted radical prostatectomy (RARP)

## Abstract

**Background:**

The pathological and oncological outcomes of retro-pubic radical prostatectomy (RRP) and robot-assisted radical prostatectomy (RARP) have not been sufficiently investigated.

**Methods:**

Treatment-naïve patients with localized prostate cancer (PC) (*n* = 908; RRP, *n* = 490; and RARP, *n* = 418) were enrolled in the study. The clinicopathological outcomes, rate and localization of the positive surgical margin (PSM), localization of PSM, and biochemical recurrence (BCR)-free survival groups were compared between RRP and RARP.

**Results:**

The median patient age and serum PSA level (ng/mL) at diagnosis were 67 years and 7.9 ng/ml, respectively, for RRP, and 67 years and 7.6 ng/ml, respectively, for RARP. The overall PSM rate with RARP was 21%, which was 11% for pT2a, 12% for pT2b, 9.8% for pT2c, 43% for pT3a, 55% for pT3b, and 0% for pT4. The overall PSM rate with RRP was 44%, which was 12% for pT2a, 18% for pT2b, 43% for pT2c, 78% for pT3a, 50% for pT3b, and 40% for pT4. The PSM rate was significantly lower for RARP in men with pT2c and pT3a (*p* < 0.0001 for both). Multivariate analysis showed that RARP reduced the risk of BCR (hazard ratio; 0.6, *p* = 0.009).

**Conclusions:**

RARP versus RRP is associated with an improved PSM rate and BCR. To examine the cancer-specific survival, further investigations are needed.

## Background

Robot-assisted radical prostatectomy (RARP) is widely used to treat localized prostate cancer (PC) [1]; nevertheless, there have been no large randomized controlled trials demonstrating its superiority over retro-pubic radical prostatectomy (RRP) [2, 3]. A recently conducted randomized controlled study that was conducted on 326 patients with localized PC, equally allocated to RARP or RRP, did not show the advantage of RARP over RRP [4]. By contrast, RARP was associated with an improved positive surgical margin (PSM) and sexual function recovery within 12 months compared to RRP in a recent meta-analysis and several comparative studies [5–7]. A study revealed its superiority in terms of the biochemical recurrence rate (BCR) at 3 years (92.1% in RRP vs 96.8% in RARP) [8], and the others performed parallel BCR between the two procedures [4, 6]. Pathological and oncological outcomes, including PSA-relapse and cancer-specific mortality, have not been sufficiently investigated.

Recently, we introduced the mentoring program during RARP, keeping the balance between surgical outcomes and surgeon education [9]. Here, we present the pathological and oncological outcomes, including localization of PSM, in men undergoing RRP and RARP at our institution.

## Methods

### Patient characteristics

Patients who underwent radical prostatectomy for localized PC between May 1, 2005 and May 31, 2016 at the University of Tokyo Hospital were included. The study was approved by the ethics committee (Permission ID: 3124) of the hospital. Written informed consent was obtained from each patient before surgery. We evaluated 908 patients with localized PC; 490 underwent RRP and 418 underwent RARP (Table 1). Patients who received any adjuvant therapy, including radiotherapy (RT) and/or androgen deprivation therapy (ADT), were excluded. Since RARP became covered by insurance in Japan in April, 2012 we have performed RARP for all patients with localized PC. Neither the type of surgical procedure performed nor the individual experience of the surgeons were taken into account in the analysis of the data. The patients were followed-up by their surgeons at 3-month intervals for 5 years and annually thereafter. Biochemical recurrence (BCR) was defined as a consecutive increase in the serum PSA level over 0.2. Some patients experiencing BCR subsequently received salvage therapy, including RT, ADT, or RT combined with ADT.

**Table 1:** Patient’s characteristics in patients with localized prostate cancer received RRP or RARP

	RRP (*n* = 490)	RARP (*n* = 418)	*P* value
Median age (ranges)	67 (51–78)	67 (47–80)	0.15
Median serum PSA (ng/mL) (ranges)	7.9 (1.3–77)	7.6 (1.4–71)	0.3
Gleason score (%)			
6	262 (54)	83 (20)	<0.0001
7	194 (40)	238 (57)	
8–10	33 (6)	97 (23)	
D’Amico classification (%)			
Low	177 (36)	62 (15)	
Intermediate	246 (50)	248 (59)	<0.0001
High	67 (14)	108 (26)	

*RRP* Retro-pubic radical prostatectomy; *RARP* Robot-assisted radical prostatectomy

### Surgical techniques

We performed RRP using the retroperitoneal approach and RARP using the peritoneal approach, as previously described [9, 10]. Cavernous nerve preservation was performed in limited patients with RRP. In RARP, cavernous nerve preservation was conducted on the cancer-negative lobe. Bilateral preservation was limited if the patient’s cancer was located at the transitional zone. Limited lymph node dissection was performed in all patients with RRP; however, it was performed in a limited number of patients who were diagnosed as having 5% or more lymph node metastasis with a Japan PC nomogram [11].

### Statistical analysis

The correlation between the age and serum PSA level was evaluated using the Wilcoxon rank sum test. The association between the clinicopathological findings and D’Amico risk classification was assessed using the chi-square test. BCR-free survival curves were plotted using the Kaplan–Meier method and verified using the Wilcoxon test. JMP 12.0 software (SAS Institute, Cary, NC) was used for the analysis, and *p* < 0.05 was considered statistically significant.

## Results

Table 1 shows the patient characteristics, including the age, serum PSA levels, Gleason score, and D’Amico risk classification [12]. The median age at diagnosis was 67 years (range, 51–78 years) for the RRP group and 67 years (range, 47–80 years) for the RARP group. The median serum PSA level at diagnosis was 7.9 ng/ml for the RRP group and 7.6 ng/ml for the RARP group. Resected specimens were evaluated by two pathologists. The Gleason scores were 6 (*n* = 262), 7 (*n* = 194), and 8–10 (*n* = 33) for the RRP group and 6 (*n* = 83), 7 (*n* = 238), and 8–10 (*n* = 97) for the RARP group. Based on the D’Amico classifications of low, intermediate, and high risk, there were 177, 246, and 67 patients, respectively, in the RRP group and 62, 248, and 108 patients, respectively, in the RARP group. Compared with the RRP group, the RARP group had significantly advanced PC, as indicated by both the Gleason score (*p* < 0.0001) and D’Amico risk classification (*p* < 0.0001).

Table 2 summarizes the pathological results, statistical analyses, and salvage therapy in the RRP and RARP groups. The RARP group was more likely to have a higher Gleason grade (*p* < 0.0001) and higher pathologic stage (*p* < 0.0001). The overall PSM rate with RARP was 21%, which was 11% for pT2a, 12% for pT2b, 9.8% for pT2c, 43% for pT3a, 55% for pT3b, and 0% for pT4. The overall PSM rate with RRP was 44%, which was 12% for pT2a, 18% for pT2b, 43% for pT2c, 78% for pT3a, 50% for pT3b, and 40% for pT4. The PSM rate was significantly lower in the RARP group of patients with stages pT2c and pT3a (*p* < 0.0001 for both). We classified the PSM site as the base, lateral lobe, apex, anterior, posterior, peri-prostatic fat tissues and seminal vesicle, as previously described [13]. Compared with the RRP group, the RARP group had PSM localization that was significantly less frequent at the lateral site (5.7% vs. 13%, *p* = 0.0003) and apex (7.8% vs. 28%, *p* = 0.0001).

**Table 2:** Pathological and oncological outcomes in men received RRP or RARP

		RRP (*n* = 490)	RARP (*n* = 418)	*P* value
Gleason score (%)	6	148 (30)	23 (5)	<0.0001
	7	295 (60)	288 (69)	
	8–10	47 (9.7)	107 (26)	
Pathological T stage (%)	0	11 (2.2)	1 (0.2)	0.007
	2a	49 (10)	43 (10)	1
	2b	82 (17)	50 (12)	0.04
	2c	219 (44)	195 (46)	0.69
	3a	118 (24)	107 (25)	0.64
	3b	6 (1)	22 (5.3)	<0.0001
	4	5 (1)	0 (0)	N.A.
Lymphovascular invasion (%)	0	390 (80)	236 (56)	<0.0001
	1	100 (20)	182 (44)	
Perineural invasion	0	203 (41)	119 (29)	<0.0001
	1	287 (59)	298 (71)	
N stage (%)	0	486 (99)	83 (93)	0.014
	1	4 (0.8)	6 (7)	
Positive surgical margin (PSM) (%)	Total	213 (44)	89 (21)	<0.0001
	pT2a	6 /49 (12)	5/43 (11)	0.92
	pT2b	15/82 (18)	6/50 (12)	0.33
	pT2c	95/219 (43)	19/195 (9.8)	<0.0001
	pT3a	92/118 (78)	46/107 (43)	<0.0001
	pT3b	3/6 (50)	13/22 (55)	0.7
	pT4	2/5 (40)	0/0	N.A.
Sites of PSM (%)	Base	33 (6.7)	32 (7.7)	0.59
	Lateral lobe	62 (13)	24 (5.7)	0.0003
	Apex	136 (28)	33 (7.8)	<0.0001
	Anterior	5 (1)	5 (1)	0.8
	Posterior	3 (0.6)	1 (0.2)	0.4
	Fat tissues	2 (0.4)	6 (1.4)	0.9
	Seminal vesicle	1 (0.2)	4 (1)	0.1
PSA-relapse (%)		121 (25)	36 (8.6)	<0.0001
Salvage therapy	RT	38	21	N.A
	ADT	61	7	
	RT + ADT	9	6	
	Surveillance	13	1	

*RRP* Retro-pubic radical prostatectomy; *RARP* Robot-assisted radical prostatectomy; *N.A.* Not applicable; *RT* radio therapy; *ADT* androgen deprivation therapy

**Fig. 1:**
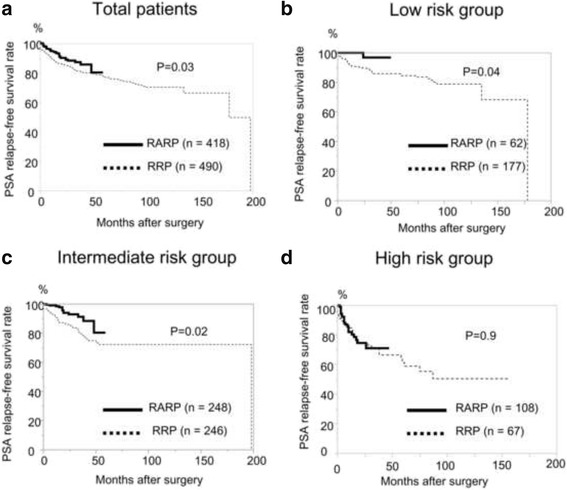
PSA relapse-free survival rates in men who underwent RARP (*black line*) and RRP (*dot line*) in total patients (**a**) and in D’Amico low (**b**), intermediate (**c**), and high group (**d**). The PSA relapse-free survival rate was significantly better in men who underwent RARP than in those who underwent RRP (A, *p* = 0.03), especially for those with a D’Amico low and intermediate risk (B and C, *p* = 0.04 and 0.02, respectively)

At the end of the follow-up period, 121 patients (25%) in the RRP group and 36 (8.7%) in the RARP group experienced BCR. The BCR-free survival rate was significantly higher in men treated with RARP than in those treated with RRP (*p* = 0.03, Fig. 1a). There were significant differences between RARP and RRP in the number of patients classified as D’Amico low risk (*p* = 0.04) and intermediate risk (*p* = 0.02) (Fig. 1b and c), but there were not significant differences in the number of patients classified as D’Amico high risk (*p* = 0.9, Fig. 1d).

Of the 108 patients classified as D’Amico high risk, 17 (17%) experienced BCR even if the resection margin was negative. Regression analyses revealed that perineural invasion was the only significant factor (*p* = 0.04) that was correlated with BCR in the D’Amico high risk patients with a negative surgical margin.

Table 3 shows the results of univariate and multivariate proportional analyses for the association of BCR-free survival rate with the surgical procedures and clinicopathological characteristics of patients. The serum PSA (*p* = 0.001), GS (*p* = 0.007), extra prostatic extension (*p* < 0.0001), lymphovascular invasion (*p* = 0.009), perineural invasion (*p* < 0.0001), resection margin (*p* < 0.0001), lymph node metastasis (*p* = 0.004), and RARP (*p* = 0.04) were all significant prognostic predictors in the univariate analysis. The serum PSA and surgical procedure were selected for the multivariate analysis because significant correlations between RARP and factors, such as the Gleason score, extra prostatic extension, lymphovascular invasion, perineural invasion, PSM, and lymph node metastasis, were observed. Lymphovascular and perineural invasion were added for the analysis according to previous studies [14, 15]. Multivariate analysis revealed that RARP (HR, 0.6), perineural invasion (HR, 1.8), and the serum PSA level (HR, 1.6) were significant prognostic predictors.

**Table 3:** Univariate and multivariate proportional hazard analyses of PSA relapse (*n* = 908)

	Univariate			Multivariate		
Variable	Hazard ratio	95% index	*p value*	Hazard ratio	95% index	*p value*
Serum PSA (ng/mL) (≥7.8 vs. <7.8)	1.7	1.2–2.3	0.001	1.6	1.2–2.2	0.005
Gleason score (≥ 8vs. ≤7)	2.0	1.4–2.9	0.007	-	-	-
Extra prostatic extension (1 vs. 0)	2.4	1.7–3.3	<0.0001	-	-	-
Lymphovascular invasion (1 vs. 0)	1.6	1.1–2.2	0.009	1.4	0.9–2.0	0.06
Perineural invasion (1 vs. 0)	2.0	1.4–2.8	<0.0001	1.8	1.3–2.7	0.001
Resection margin (1 vs. 0)	3.0	2.2–4.1	<0.0001	-	-	-
Lymph node metastasis (1 vs.0)	3.5	1.1–8.2	0.004	-	-	-
RARP vs RRP	0.7	0.4–0.9	0.04	0.6	0.4–0.9	0.009

## Discussion

We compared the pathological findings and BCR-free survival rates of patients who underwent RRP with those who underwent RARP in a consecutive series at a single institution, and we demonstrated a 40% risk reduction in BCR in patients who underwent RARP. The present study and three published studies [6, 16, 17] that compared BCR between the same groups of patients showed similar patient characteristics, including the PSM rate, PSM sites, and BCR-free survival rate, and there were comparable outcomes between RRP and RARP (Table 4). There were few possibilities for surgery selection bias because we completely switched from RRP to RARP after the instigation of insurance coverage in 2012. A propensity-based analysis to minimize treatment selection bias also demonstrated that RARP was associated with fewer PSM (13.6% vs 18.3%; odds ratio: 0.70; 95% confidence interval, 0.66―0.75) [5].

**Table 4:** Comparison studies between RRP and RRP

Author, years [ref]	Krambeck, 2009 [16]		Barocas, 2010 [17]		Park, 2014 [8]		Hu, 2014 [5]		Alemozaffar, 2015 [6]		Yaxley, 2016 [4]		Present study	
Procedure	RRP	RARP	RRP	RARP	RRP	RARP	RRP	RARP	RRP	RARP	RRP	RARP	RRP	RARP
No. of patients	588	294	491	1413	277	730	5524	5524	621	282	163	163	490	418
Age	61	61	62	61	66.8	64.2	69	69	67.2	65.4	60.4	59.6	67	67
PSA (median, range)	5.0 (0.6–39.7)	4.9 (0.5–33.5)	5.8 (4.6–8.4)	5.4 (4.3–7.4)	10.3 (−)	9.2 (−)	-	-	5.6 (−)	5.0 (−)	7.57 (4–7)	7.41 (4–10)	7.9 (1.3–77)	7.6 (1.4–71)
GS														
6 or less	391 (66.5)	192 (65.5)	221 (45.3)	723 (51.5)	54 (19.5)	167 (22.9)	1958 (35.4)	1748 (31.6)	- (62.7)	- (53)	20 (15)	23 (18)	148 (30)	23 (5)
7	167 (28.4)	87 (29.7)	213 (43.6)	588 (41.8)	159 (57.4)	458 (62.7)	2866 (51.9)	3224 (58.4)	- (28.9)	- (37)	92 (68)	87 (67)	295 (60)	288 (69)
8–10	30 (5.1)	14 (4.8)	54 (11.1)	94 (6.7)	64 (23.1)	105 (14.4)	700 (12.7)	552 (10.0)	- (8.4)	- (10)	14 (17)	20 (15)	47 (9.7)	107 (26)
PSM (%)														
Total	100 (17)	46 (15.6)	148 (30.1)	281 (19.9)	58 (20.9)	170 (23.3)	1010 (18.3)	752 (13.6)	- (23.1)	- (24.6)	15 (10)	23 (15)	213 (44)	89 (21)
pT2	-	-	-	-	7.8	11.2	3.8	2.5	-	-	2	3	33	10
pT3	-	-	-	-	36.5	44.7	18.5	13.7	-	-	12	11	76	53
pT4	-	-	-	-	-	-			-	-			40	-
Sites of PSM														
Base	10 (10)	4 (8.7)	-	-	2 (3.4)	12 (7.1)			-	-	-	-	33 (6.7)	32 (7.7)
Lateral lobe	49 (49)	27 (59)	-	-	16 (27.6)	48 (28.2)			-	-	-	-	62 (13)	24 (5.7)
Apex	56 (56)	13 (28)	-	-	22 (37.9)	58 (34.1)			-	-	-	-	136 (28)	33 (7.8)
Anterior	5 (5)	1 (2)	-	-	-	-			-	-	-	-	5 (1)	5 (1)
Posterior	-	-	-	-	-	-			-	-	-	-	3 (0.6)	1 (0.2)
Fat tissues	-	-	-	-	-	-			-	-	-	-	2 (0.4)	6 (1.4)
Seminal vesicle	-	-	-	-	-				-	-	-	-	1 (0.2)	4 (1)
Others	4 (4)	5 (11)	-	-	18 (31)	52 (30.6)			-	-	-	-		
BCR-free rate											-	-		
1y	-	-	-	-							-	-	89	94
3y	92.2	92.4	83.5	84	92.1 (pT2) 60.0 (pT3)	96.8 (pT2) 67.3 (pT3)			89.9	88.9	-	-	81	87
5y	-	-	-	-					84.7	88.0	-	-	77	80

*GS* Gleason score; *PSM* positive surgical margin; *BCR* biochemical recurrence

In our study, the PSM rates after RRP (pT2, 33%; pT3, 76%; overall, 44%) were higher than those reported in other studies [4–6, 8, 16, 17]. One possible reason was the variability in the surgical proficiency among the different surgical teams at teaching hospitals. On the other hand, because RARP was introduced using a mentoring program [9], both the oncological outcome and RARP instruction might have agreed with certified global standards. Very recently, a prospective, randomized-controlled, phase 3 study on 326 patients with localized PC allocated to either RARP or RRP by a single surgeon showed similar oncologic and functional outcomes between RARP and RRP [4]. Although it was ideal to have the same surgeon with the most expertise perform the operations to reduce surgical heterogeneity in these comparative studies, such an approach was not feasible at a teaching hospital, where several surgeons learn and perform the procedure.

The PSM sites reported in previous studies were not consistent [8, 16]. One study found significantly fewer PSM at the apex with RARP than with RRP (28% vs. 56%, *p* = 0.008) [16], which was consistent with our result. Another study did not find a significant difference in PSM at any site [8]. In this study, the significantly reduced PSM rate at the lateral site was probably due to our careful incision at the apex and lateral sites. We have several reasons for the better surgical outcomes with RARP compared RRP, especially in the apex and lateral sites. First, apical dissection could easily be performed during RARP. To prepare the dorsal vein complex (DVC), the bunching technique was used during RRP. However, this technique potentially modifies the shape of the apex or tears the prostatic capsule, resulting in tumor exposure. On the other hand, during RARP, we cut the DVC without the bunching technique. Using the DVSS scope, flexible Endowrist instruments, and careful irrigation technique, a large or complex shaped prostate in the apex could be more accurately dissected. For example, PSM was negative in patients with massive PC in the apex. Second, we dissected the lateral side of the prostate, including the peri-prostatic tissues, with the assistance of a fourth arm counter traction during RARP. This resulted in PSM reduction in pT3a cases.

Our results for the PSM rate (pT2, 10%; pT3, 53%; overall, 21%) and BCR-free survival rate (3-year, 87% and 5-year, 84%) after RARP were consistent with those of the previous studies, although our patients had PC with a high Gleason score. We found significantly better BCR-free survival rates in patients who were D’Amico low risk (3-year, 97% and 5-year, 97% for RARP vs. 3-year, 86% and 5-year, 85% for RRP) and intermediate risk (3-year, 93% and 5-year, 89% for RARP vs. 3-year, 81% and 5-year, 77% for RRP). Two recent studies also reported different BCR-free survival rates of patients according to the D’Amico risk classification [17, 18]. Barocas et al. reported BCR-free survival rates of 85% at 3 years and 80% at 5 years in an intermediate-risk group [17]. In the present study, oncological outcomes were better in patients with D’Amico low and intermediate risks, but they were not comparable to those in the high-risk group.

One reason for the high incidence of BCR in the high-risk group could be the aggressiveness of high-risk PC. Even if the surgical margin was negative, high-risk patients with peri neural invasion may be candidates for adjuvant therapy in the future. Compared with this observation, adjuvant radiotherapy improved the metastasis-free and overall survival in surgical patients with pT3N0M0 [18]. The Southwest Oncology Group showed the benefit of adjuvant androgen deprivation therapy in 481 men who were at high risk [19]. Further study is needed to clarify the oncological benefit of RARP in high-risk patients.

The present study had several limitations. First, the design was retrospective and observational at a single institution in an Asian country. Second, the follow-up period was relatively short in the RARP group. Third, the PSM may be decreased at the lateral site because there were few patients for whom the cavernous nerve was preserved compared to the patients in previous studies.

## Conclusion

We observed a better oncologic outcome in patients who underwent RARP than in those who underwent RRP at a single institution. Additional follow-up is needed to confirm the significance of these findings on PC-specific mortality.

## Abbreviations

ADT: Androgen deprivation therapy
BCR: Biochemical recurrence
DVC: Deep vein complex
PC: Prostate cancer
PSA: Prostate specific antigen
PSM: Positive surgical margin
RARP: Robot assisted radical prostatectomy
RRP: Retro pubic prostatectomy
RT: Radio therapy
